# Clinical Utility of eZIS in Cerebral Blood Flow SPECT

**DOI:** 10.3390/diagnostics15172125

**Published:** 2025-08-22

**Authors:** Shinji Yamamoto, Nobukiyo Yoshida, Noriko Sakurai, Yukinori Okada, Masayuki Satoh, Koji Takeshita, Motoki Nakai, Koichiro Abe, Mana Yoshimura, Kazuhiro Saito

**Affiliations:** 1 Department of Radiological Technology, JCHO Tokyo Yamate Medical Center, Shinjuku 169-0073, Japan; 2 Department of Radiological Technology, Faculty of Medical Technology, Niigata University of Health and Welfare, Niigata 950-3198, Japan; nobukiyo-yoshida@nuhw.ac.jp; 3 Department of Radiological Science, Faculty of Medical Science and Technology, Gunma Paz University, Takasaki 370-0006, Japan; n-sakurai@paz.ac.jp; 4 Department of Radiology, Tokyo Medical University, Tokyo 160-0023, Japan; nakai-mk@tokyo-med.ac.jp (M.N.); koichiroabe@hotmail.com (K.A.); y_mana@mac.com (M.Y.); saito-k@tokyo-med.ac.jp (K.S.); 5 Graduate School of Medical Science, Suzuka University of Medical Science, Suzuka 513-8670, Japan; 6 Center for Comprehensive Care and Research on Memory Disorders, National Center for Geriatrics and Gerontology, Obu 474-8511, Japan; bruckner@ncgg.go.jp; 7 Department of Radiology, JCHO Tokyo Yamate Medical Center, Shinjuku 169-0073, Japan; takeshita-koji@yamate.jcho.go.jp

**Keywords:** eZIS, SPECT, 99mTc-ECD, Alzheimer’s disease

## Abstract

Cerebral perfusion single-photon emission computed tomography (SPECT) is a nuclear medicine imaging technique that uses radiopharmaceuticals that selectively accumulate in the brain. However, cerebral perfusion SPECT is typically interpreted through visual assessment, making the results susceptible to observer subjectivity and varying levels of experience. The easy *Z*-score Imaging System (eZIS) is a software that quantitatively analyzes cerebral perfusion SPECT images obtained using 99mTc-ECD by comparing them with a normal database and applying *Z*-scores for quantification. The eZIS received regulatory approval in January 2015 and is currently used as an auxiliary tool for clinical diagnosis. The eZIS aids in diagnosing Alzheimer’s disease by quantifying the degree of cerebral blood flow reduction in the posterior cingulate gyrus, precuneus, and parietal lobe, which are characteristic regions affected by the disease. Additionally, it can assist in diagnosing Lewy body dementia by evaluating the “cingulate island sign,” a characteristic finding in which cerebral blood flow in the posterior cingulate gyrus and precuneus is relatively preserved compared with that in the occipital lobe. eZIS is thus extremely useful for dementia diagnosis.

## 1. Introduction

Dementia causes a significant decline in cognitive function, which impairs an individual’s ability to perform daily life activities independently [1]. Dementia can be classified as Alzheimer’s disease (AD), Lewy body dementia (LBD), or vascular dementia.

The pathology of Alzheimer’s disease is thought to involve the accumulation of amyloid-β and tau proteins in the brain [2]. In contrast, LBD is suggested to be associated with the accumulation of α-synuclein [3]. Patients with these conditions exhibit distinct clinical symptoms. A study of 770 cases of early-stage dementia (AD, vascular dementia, and frontotemporal dementia) in Japan reported that memory impairment was the initial symptom in 75.7% of the cases [4]. In contrast, in LBD, visual hallucinations occur in 61.8% of cases, whereas auditory hallucinations are observed in 30.8% of cases, demonstrating a relatively high prevalence [5].

For early cognitive impairment and early-stage AD, anti-amyloid-β antibodies, such as Lecanemab, have been reported to slow disease progression. This effect has been demonstrated in patients with early symptoms [6,7]. Early diagnosis is crucial for the use of anti-amyloid-β antibodies in AD. Furthermore, it is essential to differentiate it from other diseases that cause cognitive decline, such as LBD. LBD is characterized by visual and auditory hallucinations that serve as clues for differentiation. However, conducting a thorough medical interview can be challenging when cognitive function is significantly impaired. Therefore, objective imaging examinations are valuable for diagnosing LBD. Radiopharmaceuticals used in cerebral perfusion SPECT include 123I-IMP (123I-N-isopropyl-p-iodoamphetamine), 99mTc-HM-PAO (99mTc-hexamethylpropyleneamine oxime), and 99mTc-ECD (99mTc-ethyl cysteinate dimer). All radiopharmaceuticals are distributed in proportion to cerebral blood flow after intravenous injection. Regions with reduced cerebral blood flow appear as areas of decreased accumulation on cerebral blood flow SPECT. Analysis of the patterns of cerebral blood flow reduction has been reported to serve as an indicator of dementia.

Patients transitioning from mild cognitive impairment (MCI) to AD frequently exhibit reduced cerebral blood flow in the posterior cingulate gyrus and precuneus [8]. LBD, by contrast, is characterized by a higher frequency of cerebral blood flow reduction in the occipital lobe [9,10]. Furthermore, differences in blood flow reduction between the posterior cingulate gyrus/precuneus and the occipital lobe are useful for differentiating AD from LBD [11].

However, the subjectivity of the observer influences cerebral blood flow visual assessment using SPECT. Therefore, an objective method for evaluating the degree of cerebral blood flow reduction is essential. The easy *Z*-score Imaging System (eZIS) is a system that compares cerebral blood flow SPECT data from a participant with a database of healthy Japanese individuals using *Z*-score statistical analysis. Using eZIS enables objective and quantitative evaluation of cerebral blood flow reduction. Currently, eZIS is used as diagnostic support software for AD and LBD in routine clinical practice.

In this study, we aimed to review the characteristics of eZIS in cerebral perfusion SPECT and its underlying framework, namely 3D-SSP (three-dimensional stereotactic surface projections), and summarize its clinical and research applications.

## 2. Methods

PubMed was used to search for studies related to eZIS and other relevant publications from 2004 to 2025. The eZIS and 3D-SSP principles were referenced by citing content from textbooks published in Japan between 2004 and 2025. Permission was obtained from Kanehara & Co., Ltd. (Tokyo, Japan), Medical View Co., Ltd. (Tokyo, Japan), Nagai Shoten Co., Ltd. (Osaka, Japan), and Nanzando Co., Ltd. (Tokyo, Japan). Additionally, permission was secured from PDR Pharma Co., Ltd. (Tokyo, Japan), which is involved in developing eZIS.

A comprehensive PubMed search was conducted for studies published between 2005 and 2025, using keywords such as “eZIS,” “SPECT,” “Alzheimer’s,” and “dementia.” Articles were excluded if they had a limited number of cases (e.g., case reports), were written in Japanese or Chinese, were technical in nature, were not directly related to the research topic, did not address dementia, focused on applied research, or were a narrative review, systematic review, or meta-analysis. Finally, we excluded 12 case reports, 11 reports written in Japanese, 1 report written in Chinese, 5 technical research reports, 1 report not directly related to research, 16 reports not involved in the treatment of dementia, 3 reports on applied research, and 2 reviews.

## 3. Results

### 3.1. Fundamentals of eZIS

#### 3.1.1. What Is eZIS?

The easy *Z*-score Imaging System (eZIS) is a software designed to evaluate cerebral blood flow reductions in cerebral perfusion SPECT. It combines the advantages of 3D-SSP and Statistical Parametric Mapping (SPM) by utilizing statistical image analysis techniques. The eZIS automatically analyzes cerebral perfusion SPECT image data of the participant by comparing them with a database of normal participants and applying the *Z*-score statistical method. This process allows for an objective assessment of the cerebral blood flow reduction. eZIS supports 99mTc-ECD and 123I-IMP brain perfusion SPECT. eZIS was first developed in Japan under the leadership of Dr. Hiroshi Matsuda and is provided free of charge as eZIS Neuro by PDR Pharma Co., Ltd. It was approved as a medical device in May 2015 and has since been used in clinical settings across Japan. Initially, the eZIS was only available to support the diagnosis of AD. However, in July 2017, its use was expanded to include the diagnosis of LBD. In 2023, the newest eZIS version (Ver. 1.2.0) was released. In this version, new items were added, including reports on the dementia diagnosis flowchart from the treatment guidelines for dementia, indicating the areas of relative hyperperfusion in the brain.

eZIS contains normal databases for both 99mTc-ECD and 123I-IMP. 99mTc-ECD contains child control [1–3 years: *n* = 21; 3–5 years: *n* = 18; 6–10 years: *n* = 17; 11–15 years; *n* = 9; based on suspected disease, but with no abnormalities in brain perfusion SPECT and brain magnetic resonance imaging (MRI)] and adult control data [20–39 years: *n* = 28; 40–59 years: *n* = 30; 60–69 years: *n* = 40 (men: 18, women: 22); >70 years: *n* = 40 (men: 20, women: 20); 80-year-olds: *n* = 44 (men: 24, women: 20)]. 123I-IMP contains adult control data [20–39 years: *n* = 22; 40–59 years: *n* = 22; 60-year-olds: *n* = 22].

#### 3.1.2. Principles of 3D-SSP

Developed by Minoshima et al. [12], 3D-SSP is an analytical method for nuclear medicine brain imaging. This section provides an overview of its principles because eZIS is based on 3D-SSP. The explanation of 3D-SSP is based on the descriptions by Imahayashi [13,14,15,16,17,18], Matsuda [13,14,15,16,17,18], Mizumura [13,14,15,16,17,18], and Yamashita [18] and edited by Sasaki/Baba [19]. In 3D-SSP, the midsagittal plane is identified in the reconstructed images to correct for brain tilt during scanning. Four anatomical landmarks—the frontal pole, anterior lower margin of the corpus callosum, lower margin of the thalamus, and occipital pole—were used to align the brain to the Talairach coordinate system using the anterior–posterior commissure line. Linear transformation was applied to correct for scaling in the XYZ direction. In contrast, the nonlinear transformation was used for regional anatomical adjustments, ensuring that individual patient images matched the standard brain atlas. These corrections are performed along major neural fiber pathways, reducing the influence of brain atrophy. After transforming to the Talairach standard brain, the system measures radiotracer counts within a cortical depth of six pixels (approximately 13.5 mm) perpendicular to each pixel on the brain surface. The maximum radiotracer count at this depth is projected onto the corresponding cortical surface pixels. The extracted radiotracer count values are then normalized using reference regions such as the whole brain, cerebellum, thalamus, and pons. Next, the maximum cortical accumulation is extracted and projected onto the brain surface, minimizing anatomical misalignment in the vertical cortical direction. This process was applied to all pixels on the brain surface, and the extracted counts were normalized using a reference region. Normalized reference regions include the whole brain, thalamus, cerebellum, pons, and primary sensorimotor cortex. Whole-brain and cerebellum mean values are primarily used in normalization. The mean and standard deviation of the normalized count values are computed for each pixel and compared with those of the normal database to calculate the *Z*-score.

The *Z*-score is defined by Equation (1) as follows:

(1) $$\;Z-score=\frac{\left(mean\;voxel\;value\;\left[normal\right]-mean\;voxel\;value\;\left[patient\right]\right)}{standard\;division\;\left[normal\right]}$$

The *Z*-score represents the deviation of the imaging data of the patient from the normal mean in terms of the standard deviation. The *Z*-score reflects the degree of divergence between the average values of the participants and healthy individuals. The *Z*-score distribution is then mapped onto the brain image for visualization. The final output allows observations from eight perspectives: bilateral lateral, bilateral medial, frontal, posterior, superior, and inferior.

A normal database is constructed using volunteer participants. Each volunteer’s image undergoes the same anatomical standardization and cortical projection process as the 3D-SSP. The dataset is compiled to obtain the normalized mean and standard deviation of the pixel-wise count values to form a normal reference database. The application of 3D-SSP enables the detection of abnormalities in regions, such as the posterior cingulate gyrus and medial parietal cortex, which are otherwise difficult to evaluate visually.

#### 3.1.3. Principles and Analytical Methods of eZIS

The principles and analytical methods of eZIS were reported by Matsuda et al. in 2004 [20]. This section explains eZIS based on Matsuda’s description [14].

In eZIS, 99mTc-ECD is used as a radiopharmaceutical because it has a slow washout rate from the brain, and its clearance is independent of cerebral blood flow, making it suitable for database construction. eZIS operates without using MATLAB (MATLAB RUNTIME Library, version R2013a), which is software developed by MathWorks, Natick, MA, USA. The eZIS analysis follows the steps described below. The method for constructing a normal database also shares some of these steps [1,5] with this process.

(1) Center alignment

Brain image data were pre-aligned to the center of the imaging dataset before SPM processing to minimize errors in anatomical standardization.

(2) Morphological transformation

Original brain SPECT images were transformed to fit the Talairach standard brain template using SPM2. The images were moved to a standard brain coordinate system, followed by anatomical standardization. Linear transformation was applied to correct scaling in the XYZ direction, and nonlinear transformation was used for more detailed anatomical corrections at the local level.

(3) Smoothing and normalization

Post-standardized brain images were smoothed to correct incomplete anatomical standardization and remove noise-related inhomogeneity. An isotropic Gaussian smoothing filter with a full width at half maximum of 12 mm was applied to reduce individual differences in brain function localization, improve the signal-to-noise ratio, and ensure that the measured image values approximated a normal distribution.

(4) Masking

Scattered components from structures such as ventricles and white matter are removed.

(5) Normalization

For each normal image dataset masked for a fixed gray matter region, voxels with values exceeding one-eighth of the whole-brain voxel average or the average count of the cerebellar hemisphere with the highest activity were used for normalization. This process generated mean and standard deviation images for each voxel.

(6) *Z*-score calculation

The corrected normal image database was statistically compared with the individual images on a voxel-by-voxel basis, and *Z*-scores were displayed in the transverse, sagittal, and coronal planes. A *Z*-score map of the transverse plane was generated. Among the *Z*-scores up to 14 mm in depth from the cortical surface, those exceeding a predefined threshold were averaged and displayed as cortical values. The direction of the cortical surface normal was estimated from the 27 neighboring pixels, including the target cortical pixels. The *Z*-score represents the deviation of the image of the participant from the normal mean, expressed in units of standard deviation using Equation (1). A *Z*-score of two indicates that the value of the participant exceeded two standard deviations from the mean, corresponding to a 5% level of statistical significance. In other words, *Z*-scores > 2 correspond to *p* < 0.05.

### 3.2. Diagnosis of Alzheimer’s Disease Using eZIS

#### 3.2.1. Determination of Cutoff Values in eZIS

Diagnosis of AD using cerebral blood flow SPECT is generally performed through visual assessment. However, visual assessment relies on the observer’s subjectivity, making it necessary to establish reference values (cutoff values) for an objective and automated diagnosis. eZIS enables the estimation of the likelihood of AD using quantitative indices. Three key parameters are used in this process: (1) severity of blood flow reduction in disease-specific regions, (2) percentage of blood flow reduction in disease-specific regions, and (3) ratio of blood flow reduction in disease-specific regions to whole-brain reduction. eZIS designates the posterior cingulate gyrus, precuneus, and parietal lobe as disease-specific regions to diagnose Alzheimer’s.

(1) Severity of blood flow reduction in the disease-specific regions (severity)

This index indicates the degree of blood flow reduction in the regions affected by AD. In the range of 0–1, there was little to no decrease in blood flow in the disease-specific regions. A slight reduction in blood flow was observed in the range of 1–2. A significant decrease was noted in the range of 2 to 3; at 3 or higher, the reduction in blood flow within the disease-specific regions was considered substantial.

(2) Percentage of areas with blood flow reduction in disease-specific regions (extent)

The extent parameter represents the proportion of regions within the disease-specific area where the *Z*-score is 2 or higher. In other words, it indicates the extent of regions within the disease-specific area that show a significant reduction in blood flow compared with healthy individuals.

(3) Ratio of blood flow reduction in disease-specific regions to whole-brain reduction (ratio)

The ratio is defined as the proportion of regions within the disease-specific area where the *Z*-score is 2 or higher, relative to the proportion of regions in the whole brain where the *Z*-score is 2 or higher. In other words, it represents the proportion of blood flow reduction areas in the whole brain located within the disease-specific region. It serves as an indicator of the specificity of blood flow reduction within the disease-specific area.

Additionally, regarding the cutoff values, the following were determined:

(1) Severity of blood flow reduction in the disease-specific regions (severity)

Cutoff Value: 1.2.

(2) Percentage of areas with blood flow reduction in disease-specific regions (extent)

Cutoff Value: 14.2%.

(3) Ratio of blood flow reduction in disease-specific regions to whole-brain reduction (ratio)

Cutoff Value: 2.22.

If all three parameters exceeded the cutoff values, the likelihood of Alzheimer’s disease was considered high. These cutoff values were determined based on a study comparing 40 patients with AD and 40 healthy individuals [21]. When the cutoff value for severity was set at 1.19, the area under the curve (AUC) was 0.924. With a cutoff value of 14.2%, the AUC was 0.934. For the ratio with a cutoff value of 2.22, the AUC was 0.862. Additionally, the diagnostic accuracies for very early disease have been reported to be 85%, 86%, and 80%. Among 201 patients—including 12 with AD, 9 with vascular dementia, and 9 without dementia—the sensitivity and specificity were 100% and 45% for discriminating AD from non-dementia using a severity cutoff value of 1.19 [22]. For a cutoff value of 14.2 for extent, both sensitivity and specificity were 100%. For a ratio cutoff of 2.22, the sensitivity and specificity were 42% and 100%, respectively [22]. In a group of 86 MCI patients (progressive MCI: *n* = 40, age: 72.6 years, 18 males; stable MCI: *n* = 46, age: 71.0 years, 17 males), the eZIS scores for predicting the conversion from MCI to AD were as follows: for severity (cutoff value: 1.22), AUC = 0.719, sensitivity = 0.700, and specificity = 0.696; for extent (cutoff value: 10.475), AUC = 0.712, sensitivity = 0.725, and specificity = 0.696; and for ratio (cutoff value: 1.005), AUC = 0.698, sensitivity = 0.850, and specificity = 0.522 [23].

If all three parameters exceeded the cutoff values, the likelihood of Alzheimer’s disease was considered high. These cutoff values were determined based on a study comparing 40 patients with AD and 40 healthy individuals [20]. When the cutoff value for severity was set at 1.19, the area under the curve (AUC) was 0.924. With a cutoff value of 14.2%, the AUC was 0.934. For the ratio with a cutoff value of 2.22, the AUC was 0.862 (Table 1).

#### 3.2.2. Studies on Alzheimer’s Disease and Mild Cognitive Impairment (MCI) Using eZIS

- Comparison with Clinicopathological Findings

A comparison was made between 13 cases (3 males, 10 females, age 78.2 ± 5.8 years) that were pathologically determined not to have AD and 24 cases (8 males, 16 females, age 76.6 ± 3.9 years) that were pathologically diagnosed with AD. The results showed that severity was 1.21 ± 0.32 in the non-AD group and 1.55 ± 0.57 in the AD group, extent was 13.33 ± 12.2 in the non-AD group and 25.5 ± 18.9 in the AD group, and ratio was 1.69 ± 1.38 in the non-AD group and 2.94 ± 1.93 in the AD group. All the indices were higher in the AD group [24].

- Comparison with Visual Evaluation

A study was conducted using brain perfusion SPECT to assess diagnostic differentiation in patients with AD, vascular dementia (VaD), AD with vascular dementia (AD + VaD), mixed dementia, frontotemporal dementia (FTD), and DLB. The study included 23 AD cases (4 males, 19 females, 82.1 ± 5.2 years), 20 AD + VaD cases (4 males, 16 females, 82.4 ± 4.9 years), 5 mixed dementia cases (1 male, 4 females, 85.4 ± 2.5 years), 31 VaD cases (10 males, 21 females, 80.6 ± 5.3 years), 7 FTD cases (2 males, 5 females, 79.6 ± 5.5 years), and 3 DLB cases (3 females, 82.3 ± 2.4 years).

For diagnosing AD, AD + VaD, and mixed dementia, visual evaluation alone showed a sensitivity of 33.3%, a specificity of 73.2%, and an accuracy of 51.7%. Using eZIS alone (with a cutoff of 14.2% for the disease-specific blood flow reduction area), the sensitivity, specificity, and accuracy were 39.6%, 82.9%, and 59.6%, respectively. When visual evaluation was combined with eZIS, sensitivity increased to 41.7%, specificity to 85.4%, and accuracy to 61.8%.

Additionally, when using the SMH sign (sensorimotor hot spot = increased accumulation in the sensorimotor cortex) on SPECT, the SMH sign alone yielded a sensitivity of 58.3%, specificity of 75.6%, and accuracy of 66.3%. Combining visual evaluation with the SMH sign resulted in a sensitivity, specificity, and accuracy of 64.5%, 63.4%, and 64.0%, respectively. A comprehensive evaluation combining visual assessment, eZIS, and the SMH sign further improved the sensitivity to 70.8%, specificity to 68.3%, and accuracy to 69.7% [25].

The results are presented in Table 2.

- Comparison with amyloid PET

A study was conducted among 10 male and 13 female patients with MCI (mean age: 74.2 years). The analysis included 12 patients with positive accumulation of 11C-Pittsburgh Compound B (11C-PIB), which reflects amyloid deposition in the brain, and 11 patients with negative accumulation.

Using the 99mTc-ECD brain perfusion SPECT and three eZIS scores to evaluate the presence or absence of abnormalities, the severity parameter revealed five (41.7%) and three patients (27.3%) with and without 11C-PIB, respectively. The extent parameter revealed that three patients (25.0%) demonstrated accumulation, and three (27.3%) did not. The ratio parameter revealed that five patients (41.3%) exhibited accumulation, and zero cases (0%) did not. Notably, 11C-PIB accumulation was not observed when the ratio parameter was within the reference range [26].

The results are presented in Table 3.

### 3.3. Diagnosis of Dementia with Lewy Bodies Using eZIS

#### 3.3.1. Diagnosis of the Cingulate Island Sign Using 18F-FDG

The cingulate island sign is a characteristic finding in DLB, indicating relatively preserved 18F-FDG uptake in the posterior cingulate gyrus. 18F-FDG PET is a useful marker for distinguishing between AD and DLB [27].

The CIS ratio was defined as follows: (18F-FDG PET uptake in the posterior cingulate cortex)/(18F-FDG PET uptake in the precuneus + cuneus) [28].

A study using 18F-FDG PET was conducted on 22 patients with DLB and 26 patients with amyloid-positive AD. Differentiation between these two conditions based on the CIS was assessed using the Mini-Mental State Examination (MMSE) [29]. The MMSE is a 30-point cognitive test, where scores of 28–30 indicate normal cognition, 24–27 suggest mild cognitive impairment (MCI), and 23 or below suggest dementia.

The sensitivity and specificity of the CIS-based differentiation for patients with MMSE scores > 24 were 66.7% and 77.8%, respectively. For patients with MMSE scores between 20 and 24, the sensitivity and specificity were 91.7% and 100%, respectively, demonstrating a high diagnostic accuracy. In patients with MMSE scores < 20, the sensitivity and specificity were 75.0% and 66.7% [30].

#### 3.3.2. Diagnosis of the Cingulate Island Sign Using Brain Perfusion SPECT

The CIS has also been used for brain perfusion SPECT. A study using 99mTc-ECD brain perfusion SPECT compared 17 cases of probable DLB and 18 cases of amyloid-positive AD. The results showed that the AUC for posterior cingulate and medial occipital hypoperfusion was 0.614, and lateral occipital hypoperfusion was 0.716, 0.614, and 0.585, respectively. The diagnostic accuracy of the CIS score was 85.7%, with a sensitivity of 88.9%, specificity of 82.4%, and AUC of 0.87 [31].

These findings indicate that in DLB, blood flow in the posterior cingulate cortex is relatively preserved compared to that in the occipital lobe, a characteristic feature of the disease. The cingulate island sign (CIS) is also observed in brain perfusion SPECT, enabling differentiation between AD and DLB. The CIS score is used in the eZIS and is defined by the following equation (Equation (2)):

(2) $$CIS\;score=\frac{Total\;Z-score\;for\;decreased\;brain\;perfusion\;at\;posterior\;sin\;grate\;gyrus}{Total\;Z-score\;for\;decreased\;brain\;perfusion\;at\;occipital\;lobe}$$

When the CIS score is 0.281 or higher, the likelihood of DLB is low, whereas when it is below 0.281, the possibility of DLB is considered. The diagnostic accuracy of distinguishing AD from DLB using the CIS score has been reported to be 84.6%, with a sensitivity of 92.3% and a specificity of 76.9% [32].

**Figure 1:**
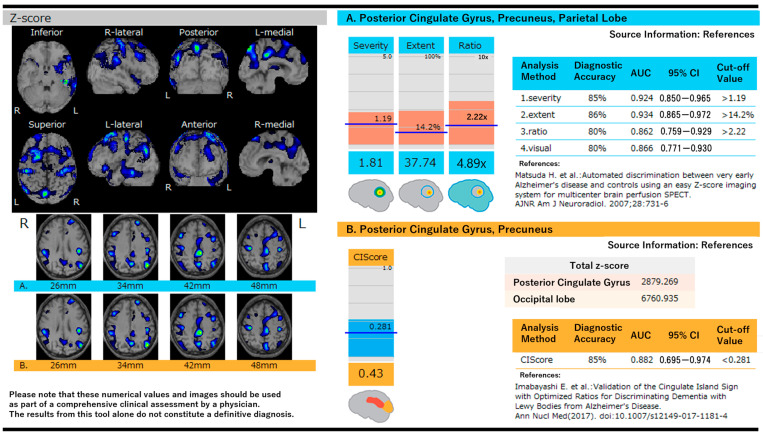
Score analysis in Alzheimer’s disease and dementia with Lewy bodies using eZIS and *Z*-score mapping on the cortical surface. (**A**) *Z*-score maps are shown on cortical surface projections with color-coded regions representing significant reductions in cerebral blood flow. Quantitative indices include severity, extent, and ratio, which assess the degree and distribution of hypoperfusion in AD-characteristic regions [21]. (**B**) The cingulate island sign (CIS) score is used to differentiate DLB from AD by comparing *Z*-scores in the posterior cingulate and occipital lobes. A CIS score < 0.281 is indicative of DLB. Diagnostic performance metrics, including AUC, sensitivity, and specificity, are shown for both scoring systems [32].

Figure 1 illustrates the score analysis of AD and DLB using eZIS, along with the *Z*-score mapping of the cortical surface. Table 4 presents the AUC and diagnostic performance of eZIS for diagnosing DLB.

However, among patients with DLB, in the normal (*n* = 17; men: 8; women: 9) and abnormal CIS score groups (*n* = 26; men: 13; women: 13), the mean ages were 82.2 ± 5.1 and 75.7 ± 5.8 years, respectively, and the frequency of CIS score was lower in the high-age patient group, especially those aged > 79 years [33].

Among the patients with MCI (*n* = 14), AD (*n* = 14), and DLB, a CIS score threshold value of 0.225 yielded an AUC of 0.941, with a sensitivity of 87.5% and specificity of 93.4% for the differential diagnosis between AD and DLB [34].

## 4. Conclusions

99mTc-ECD brain perfusion SPECT data can be compared with a database of healthy individuals using the eZIS. This comparison utilizes the *Z*-score, in which regions with a *Z*-score of two or higher indicate statistically significant hypoperfusion. Using the *Z*-score enables an objective assessment of the degree of cerebral blood flow reduction. When the posterior cingulate cortex, precuneus, and parietal lobe are used as disease-specific regions, and the participant’s eZIS scores exceed all three of the following cutoff values—1.2 for severity (degree of hypoperfusion in disease-specific regions), 14.2% for extent (proportion of hypoperfused areas within disease-specific regions), and 2.22 for ratio (proportion of hypoperfused areas in disease-specific regions relative to the whole brain)—AD is suggested. However, if the CIS score is below 0.281, DLB is suggested.

### Limitations

Positron emission tomography (PET) with amyloid-binding tracers has also been employed in diagnosing dementia. However, including this modality would broaden the study’s scope and deviate from this investigation’s primary objective; therefore, it was not considered.

## Figures and Tables

**Table 1:** Cutoff values and diagnostic accuracy for Alzheimer’s disease diagnosis using eZIS.

Factor	Cutoff Value	AUC	Diagnostic Accuracy for Early-State Alzheimer’s Disease
Severity	1.2	0.924	85%
Extent	14.2%	0.934	86%
Ratio	2.2	0.862	80%

AUC: area under the curve.

**Table 2:** Diagnostic accuracy of visual evaluation, eZIS, SMH sign, and their combinations.

Diagnostic Method	Sensitivity	Specificity	Accuracy
Visual assessment only	33.30%	73.20%	51.70%
eZIS only	39.60%	82.90%	59.60%
Visual assessment + eZIS	41.70%	85.40%	61.80%
SMH sign	58.30%	75.60%	66.30%
Visual assessment + SMH sign	64.50%	63.40%	64.00%
Combination of SMH signs	70.80%	68.30%	69.70%

SMH = sensorimotor hot spot sign.

**Table 3:** 11C-PIB uptake and eZIS score.

Score	^11^C-PIB Positive	^11^C-PIB Negative
Severity	5 Cases (41.7%)	3 Cases (27.3%)
Extent	3 Cases (25.0%)	3 Cases (27.3%)
Ratio	5 Cases (41.3%)	0 Cases (0%)

**Table 4:** AUC and diagnostic accuracy of Lewy body dementia in eZIS.

Factor	AUC	Diagnostic Accuracy
Blood flow reduction in the posterior cingulate gyrus	0.716	
Blood flow reduction in the medial occipital lobe	0.614	
Blood flow reduction in the lateral occipital lobe	0.585	
CIS	0.870	Accuracy: 85.7%; sensitivity: 88.9%; specificity: 82.4% [32] Accuracy: 84.6%; sensitivity: 92.3%; specificity: 76.9% [33]

AUC = area under the curve; CIS = cingulate island sign.
